# Exploring How Adipose Tissue, Obesity, and Gender Influence the Immune Response to Vaccines: A Comprehensive Narrative Review

**DOI:** 10.3390/ijms26020862

**Published:** 2025-01-20

**Authors:** Juan Bautista De Sanctis, Germán Balda Noria, Alexis Hipólito García

**Affiliations:** 1 Institute of Molecular and Translational Medicine, Faculty of Medicine and Dentistry, Palacky University, Hněvotínská 1333/5, 77900 Olomouc, Czech Republic; 2 Czech Advanced Technology and Research Institute, Palacky University, 77900 Olomouc, Czech Republic; 3 Institute of Immunology Nicolás Enrique Bianco, Faculty of Medicine, Universidad Central de Venezuela Los Chaguaramos, Caracas 1040, Venezuela; germanbalda.med@gmail.com

**Keywords:** gender, obesity, immune response, adipokines, thyroid hormones, vaccine response, inactivated vaccine, recombinant vaccines, mRNA vaccines

## Abstract

Vaccines represent an essential tool for the prevention of infectious diseases. Upon administration, a complex interaction occurs between the vaccine formulation and the recipient’s immune system, ultimately resulting in protection against disease. Significant variability exists in individual and population responses to vaccination, and these differences remain the focus of the ongoing research. Notably, well-documented factors, such as age, gender, and genetic predisposition, influence immune responses. In contrast, the effects of overweight and obesity have not been as thoroughly investigated. The evidence indicates that a high body mass index (BMI) constitutes a significant risk factor for infections in general, with adipose tissue playing a crucial role in modulating the immune response. Furthermore, suboptimal levels of vaccine seroconversion have been observed among individuals with obesity. This review provides a plausible examination of the immunity and protection conferred by various vaccines in individuals with an overweight status, offering a comprehensive analysis of the mechanisms to enhance vaccination efficiency.

## 1. Introduction

The response to vaccination constitutes a multifaceted phenomenon that necessitates the proper activation of the immune response, facilitating an adequate defense against infection or disease [1]. In recent years, particularly during the vaccination campaign against the SARS-CoV-2 virus, the issues of overweight and obesity have gathered significant attention due to observations indicating that individuals with obesity have an increased risk of severe disease [2,3] and may exhibit a diminished response to vaccines [4,5,6]. Nonetheless, the literature presents conflicting reports on obesity and vaccine response, highlighting the necessity for a comprehensive understanding of the underlying mechanisms [4,5,6,7]. This article seeks to review the relationship between adipose tissue, obesity, and the immune response, examine the existing vaccine literature, and explore the potential mechanisms implicated in this phenomenon.

## 2. Overview of Adipose Tissue Physiology and Physiopathology

**Figure 1:**
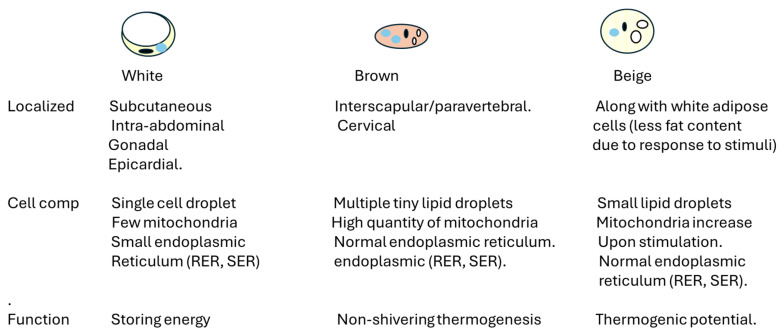
The figure represents the characteristics of the different adipose cells. The white adipose cells contain the highest amount of stored lipids and are low responders to stimuli; the brown adipose cells are involved in thermogenesis to control body temperature; and the beige adipose cells also serve as lipid deposits, but control temperature upon stimulation. Beige adipose cells are found along white adipose cells in the adipose tissue. Cell comp refers to organelle cell composition. The white circles refer to lipid deposits, the blue circles refer to mitochondria, and the black circles refer to the nucleus.

Adipose tissue is a connective tissue characterized by the absence of fibroblasts and the minimal presence of fibrous structures [8]. It falls under the category of loose connective tissue instead of dense connective tissue [8,9]. Its primary cellular component is adipocytes, which store triglycerides crucial for energy metabolism. Adipocytes are the primary cell type found in adipose tissue [8,9,10]. When energy is abundant, they store it as lipid droplets and release it when the body requires it [8,9,10]. Figure 1 describes the different types of adipose cells and their location. Most scholars categorize body fat based on the predominant effects on overall health, offering a coherent framework for understanding their implications. It has been shown that the response to stimuli, the beta-adrenergic response, differs among the different adipocytes. Abdominal adipocytes are more responsive to the lipolytic action of beta 1-adrenergic agonists. In contrast, gluteal adipocytes are more responsive to the antilipolytic action of alpha 2-adrenergic agonists, and beta-3 adrenergic receptors are involved in brown adipose tissue activation [8,9,10]. Moreover, non-shivering thermogenesis is a process that generates heat to prevent hypothermia without the need for muscle shivering. This mechanism is activated by cold exposure and also aids in maintaining energy balance by dissipating excess heat [8,9,10]. Thyroid hormones maintain energy balance and are implicated in thermogenesis [8,9,10]. Thus, depending on the location, the response of adipocytes and adipose tissue differs, as shown in Figure 1.

Statistically, women tend to possess a higher percentage of adipose tissue than men, with a tendency to accumulate fat in the subcutaneous layer [11]. In contrast, men often exhibit an accumulation of adipose tissue in the abdominal region, particularly within the visceral compartment [11]. Visceral fat is correlated with an increased risk of developing metabolic diseases.

Beyond their role in energy storage, adipocytes also have endocrine functions. They secrete a variety of adipokines, which are crucial for the homeostasis of adipose tissue and the link between adipose cells and the immune cells within the tissue. Table 1 summarizes the most relevant adipokines studied in murine models and humans [12,13,14,15,16,17,18,19,20,21,22,23,24,25,26].

Other intermediates involved in adipose tissue physiological and physio-pathological responses have been identified as playing a significant role in the physiological responses of adipose tissue (Table 2). Some of these intermediates have been hypothesized to function as adipokines; however, discrepancies remain in this area of research. In murine models, the role of these intermediates has been analyzed and validated using normal analysis or knockout models. However, in humans, most of the analysis has been validated indirectly, either by measuring soluble intermediates (cytokines and receptor antagonists) or by pharmacological inhibition, as is the case of dipeptidyl peptidase 4, retinol binding protein 4.

The role of adipokines and related factors are critical to adipocyte and adipose tissue responses. However, these factors are not independent of other processes (endocrine, immune cell migration, and others) in the complex process of adipose tissue homeostasis.

### 2.1. Adipose Tissue, Gender, and Immune Response

There is an increased prevalence of overweight and obese women [44]. Gender differences are observed in the prevalence and phenotype of obesity, body fat distribution, drug efficacy, representation in clinical trials, and the varied secondary effects associated with bariatric surgery. Hence, gender emerges as a critical variable in the analysis of obesity [45].

Sex hormones significantly influence gender differences in body composition. The research indicates that women typically exhibit better insulin sensitivity despite having higher adiposity levels than men [46]. This phenomenon may be related to decreased insulin sensitivity observed after menopause, with estrogen therapy shown to enhance insulin sensitivity [45,46]. Furthermore, androgens have distinct effects on adipose tissue and insulin resistance that vary between the sexes [45,46]. Elevated androgen levels in women correlate with increased insulin resistance, while lower testosterone levels in men are linked to insulin resistance; this condition tends to improve with testosterone replacement therapy [46]. The levels of adipokines also differ between genders [47,48,49], and these variations are associated with the risk of developing type 2 diabetes [50]. It is important to note that type 2 diabetes is a chronic metabolic disease, and its effects on adipose tissue and immune response are complex.

Obesity has also been associated with the aging process due to an increased subclinical inflammatory response; however, variations in hormonal conditions may lead to differing experiences of aging between genders [51]. Estrogen is particularly linked to a preferential increase in subcutaneous adipose tissue, as opposed to visceral adipose tissue, primarily induced by testosterone. This distinction also correlates with the risk of various associated diseases, notably cardiovascular conditions and cancer [51]. Despite this indirect evidence, it is imperative to recognize the significance of the hypothalamus–pituitary–adrenal axis [48]. This axis is critical not only for the regulation of sex hormones but also for the management of adipokines that play a vital role in maintaining adipose tissue homeostasis and growth.

Luo and coworkers [52] analyzed a population of children and adolescents, finding that the prevalence of inflammatory markers was higher in overweight and obese individuals. They also observed gender differences. However, the difference was less prevalent in the adolescent population. Silva and Iwasaki [53] summarized published data that post-acute infection syndromes are prevalent in females.

A recent bioinformatic analysis [54] revealed that chromosome interactions in the Eurasian admixed population showed that the X chromosome acted on autosomal immunity-associated genes. Consequently, the immune response of admixed populations should not differ between ethnic groups. However, Persons and coworkers [55] showed differences in obesity among different races in the USA.

**Figure 2:**
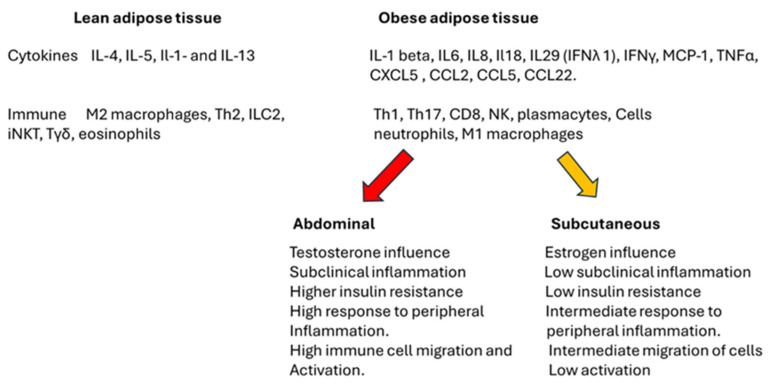
The distinct differences between lean and obese adipose tissue. The obese tissue is characterized by the loss of a tolerogenic environment and the infiltration of immune cells. In adipose tissue, the inflammatory cytokines are more prevalent than the tolerogenic cytokines present in lean adipose tissue. The inflammatory response also involves pro-inflammatory cells as described in obese tissue as compared to the tolerogenic cells in normal adipose tissue. The scheme also presents the general impact of testosterone and estrogen depending on the site in which adipose tissue is located. Subcutaneous adipose tissue, controlled mainly by female hormones, differs from abdominal tissue in the low involvement in insulin resistance and local and peripheral inflammation. An increase in abdominal adipose tissue has been linked to cardiovascular diseases and diabetes.

Figure 2 delineates the distinctions between lean and obese adipose tissue, focusing on the role of immune cells, cytokines, and sex hormones associated with visceral and subcutaneous adipose tissue. It is essential to highlight that, notwithstanding the variations presented in the figure, stable adipose tissue may exhibit immunological tolerance regardless of its size. However, under conditions of stress induced by peripheral or localized factors, the cells and cytokines can provoke an inflammatory response within the tissue. This response may result in the deregulation of metabolic processes and peripheral function, ultimately contributing to the development of insulin resistance and an exacerbated inflammatory response.

Numerous authors have documented variations in the immune response based on sex [56,57]. However, the mechanisms underlying specific responses remain undefined, and some researchers inappropriately extrapolate the findings from rodent studies to humans. Popotas and colleagues [58] examined Toll-like receptors (TLRs) as potential mechanisms for elucidating responses to pathogens and other stimuli. Their rationale is grounded in the fact that the X chromosome influences TLR regulation [58]. Notably, there are disparities in TLR receptor expression and functionality when comparing humans and mice. Specifically, TLR7 and TLR8 are expressed at higher levels in female immune cells than in male cells, while TLR4 exhibits the opposite pattern [58]. Furthermore, TLR9 signaling is significantly elevated in females, correlating with increased production of interferon types I and II [58]. It can be proposed that the immune response may depend on the initial signaling that induces cellular activation. Further research is warranted to explore this matter comprehensively.

Recently, Wang P et al. showed in a murine model that TLR9 deficiency leads to obesity [59]. This receptor has also been associated with autoimmune diseases [60]. How obesity, gender, and autoimmune diseases are connected is still under investigation.

Layug and coworkers [61] also reviewed the difference in CD8+ lymphocyte response, showing that females have more CD8+ effector and memory lymphocytes to respond to pathogens. In addition, the CD8+ lymphocyte response in males with autoimmunity and cancer is characterized by cell exhaustion compared to the females, in which the effector cells are responsible for the process. Forsyth and coworkers partially share the proposal since they envision the responses to the genetic impact of the X chromosome [62]. Several points on the X chromosome have been involved in innate and adaptative immune responses, and, according to the authors [62], these events are responsible for the increased susceptibility of males to infections. There are still many enigmas to solve in the puzzle. Still, it can be concluded that gender, sex hormones, and adipose tissue endocrine functions influence the efficiency of the immune response.

### 2.2. Thyroid Hormones, Gender, and Immune Response

A recent review conducted by Hoffmann and colleagues [63] indicates that sex hormones influence immune cell responses by altering both cell function and migration. The authors assert that specific receptors, primarily evaluated through pharmacological agonists, antagonists, or murine knockout models, are crucial for defining these immune cell responses.

On the other hand, hypothyroidism has been related to weight gain and obesity [64]. Hyperthyrotropinemia associated with obesity may also lead to an increased susceptibility to thyroid autoimmunity and subsequent hypothyroidism [64]. Elevated levels of leptin may contribute to the hyperthyrotropinemia observed in obesity and may also heighten the risk of developing thyroid autoimmunity, potentially leading to subsequent hypothyroidism [64]. Levothyroxine treatment has a limited effect on obesity [65]. The effect of levothyroxine treatment in subclinical hypothyroidism and sex hormone production has not been well described. According to Sror-Turkel et al. [66], low TSH and T3 are good prognostic of mortality in patients with severe COVID-19 infection [66]. In addition, vaccines against SARS-CoV-2 have been linked to thyroid dysfunction [67].

Autoimmune thyroiditis is more prevalent in women than in men [68]. Although most autoimmune disorders have a genetic background, viral infection has been related to this autoimmune disorder [68]. Similarly, treatment with checkpoint inhibitors generates thyroid dysfunction, and levothyroxine partially restores thyroid function and immune response [69].

Reduced thyroid hormone levels are directly associated with decreased estrogen and androgen production [70]. In contrast, high levels of thyroid-stimulating hormone, commonly observed in hypothyroidism, are frequently correlated with increased prolactin levels [70]. This association may result in a delayed luteinizing hormone (LH) response and lead to irregularities in ovulation and spermatogenesis [70]. Elevated prolactin levels impact various cellular responses, including those of the immune system [70]. Conversely, progesterone promotes thyroid hormone secretion, establishing a reciprocal relationship between these hormones. It can be concluded that an isolated evaluation of sex hormones in the context of immune responses presupposes normal prolactin levels and normal thyroid hormone function. To address this issue, Table 3 compares the effects of the sex hormones and thyroid hormones on the immune response.

The relationship between obesity and the risk of autoimmune thyroid dysfunction (more prevalent in females), which serves as the primary cause of hypothyroidism in adults, remains an area of considerable uncertainty [70,81]. The studies indicate that the prevalence of autoimmune thyroid dysfunction among individuals with obesity is approximately 10% in the pediatric population and varies between 10% and 60% in adult populations [82]. A study in the USA reported a positive association between BMI and waist circumference with serum TSH and T(3) levels but not fT(4) in euthyroid adults [83], and the increase in obesity was linked to a decrease in sexual function. Consequently, thyroid hormones are a critical link between hormone dysfunction and metabolic changes in overweight and obesity.

## 3. Adipocytes as Antigen-Presenting Cells

Recent research has highlighted the involvement of adipocytes in immune responses, as they can recruit and activate immune cells [84,85]. They are antigen-presenting cells (APCs) expressing CD1d and MHC class I and II molecules [86,87]. Adipocytes can directly activate CD4+ T lymphocytes through the antigen: the MHCII complex in a contact-dependent manner [86,87]. A recent study has shown that adipocytes also express MHC class II molecules, along with co-stimulatory molecules CD80 and CD86, and their expression is significantly heightened in response to high-fat diets [88]. While adipocytes display MHC class I molecules like other nucleated cells, there is still inconclusive evidence regarding direct interactions with CD8+ T lymphocytes via the antigen: the MHCI complex [89]. Conversely, studies have demonstrated that CD1d expressed in adipocytes can present lipid antigens to invariant natural killer T (iNKT) cells, effectively stimulating their activation [90,91].

In obesity, both local and systemic immune dysfunctions arise from metabolic stress [92]. In adipose tissue, the immune cells that are normally anti-inflammatory and immune-regulatory—such as M2-type macrophages, regulatory T cells (Tregs), Th2, and type 2 innate lymphoid cells (ILC2s)—are replaced by a higher number of pro-inflammatory immune cells. These include M1 macrophages, Th1, Th17, Th22, and CD8+ T lymphocytes, which secrete pro-inflammatory cytokines, like IL-1β, IL-6, IL-17, and IFN-γ [93,94]. This pro-inflammatory response may be exacerbated by intestinal inflammation associated with obesity [95]. In addition to the local immune changes within adipose tissue, systemic immune adaptations are also evident in obesity, characterized by increased circulating numbers of monocytes, neutrophils, and lymphocytes (Th1, Th17, and Th22), along with a decrease in circulating Treg lymphocytes and elevated levels of pro-inflammatory cytokines [93,94,95]. Collectively, these alterations create a pro-inflammatory state of the immune system in obese individuals, marked by heightened cytokine levels both locally in adipose tissue and systemically [93]. This chronically elevated inflammatory condition is believed to stimulate regulatory pathways that ultimately restrict the immune response to acute infections. A notable example is the compromised type I interferon antiviral response observed in individuals with obesity [31].

Table 4 illustrates the different cells directly and indirectly involved in adipose tissue physiology and physiopathology. The difference with Figure 2 derives from a comprehensive analysis of all the possible cells described in the white adipose tissue increase, and stress response is present [96,97,98,99,100,101,102,103,104,105,106,107,108,109,110,111,112,113,114,115,116,117,118,119,120,121,122,123]. It is important to note that the role of mesenchymal stem cells in tissue repair and remodeling is recent and is still under investigation, as well as the possible role of follicular B and T cells in the link between lymphoid organs, leukocyte migration, immune response, inflammation, and autoimmunity [124].

The mechanisms by which leptin exerts its effects on immune cells are complex, partially due to the presence of multiple isoforms of the leptin receptor generated through alternative splicing, each with distinct signaling capabilities [125]. For example, T lymphocytes predominantly express the long form of the leptin receptor, particularly following activation, whereas neutrophils primarily express the short form. On the other hand, NK cells express short- and long-form receptors. Individuals with genetic mutations that impair the synthesis of leptin are often morbidly obese and exhibit compromised immune defenses [125]. Obesity leads to hyperleptinemia, which can adversely affect the immune response [125,126]. Moreover, obesity has been associated with increased thymic senescence and a reduction in the diversity of the T-cell repertoire, potentially impacting immune surveillance [125,126]. Numerous studies, reviewed by Muscogiuri and coworkers [45], have highlighted that obesity constitutes a significant risk factor for postoperative and surgical nosocomial infections.

Deng and colleagues [127] showed that low serum leptin levels in young and elderly healthy subjects are associated with lower antibody responses to influenza and hepatitis B (HBV) vaccines. Leptin stimulates the differentiation and function of human and mouse TFH cells in culture and is also required to maintain TFH function and sustained effective humoral immunity [127]. TFH is necessary to support and maintain effective humoral immunity to infection and immunization in mice. The mechanism of action of leptin is regulated in part by activation of the Stat3 and mTOR (mechanistic target of rapamycin) pathways [127]. Their results suggest that leptin is a physiological regulator of TFH function and that leptin deficiency may serve as a biomarker to identify the risk of low vaccine efficacy. Moreover, serum leptin levels did not always correlate positively with absolute antibody titers after vaccination or changes in antibody titers in adults vaccinated against influenza or HBV. Overall, their data [127] support the notion that leptin is a natural regulator of TFH cells in the general population. This should not be interpreted to mean that higher levels of leptin are associated with higher vaccine responses; on the contrary, in their view, leptin constitutes a metabolically mediated threshold factor that is needed to mount normal vaccine responses.

Investigating the inflammatory response within adipose tissue presents a complex scenario [128,129,130]. The infiltration of various cell types into this tissue, driven by metabolic demands or stressors, increases chemokine production [128,129,130]. This increase in chemokines subsequently facilitates the migration of cells, thereby fostering an inflammatory environment [129]. The activation of macrophages by external stimuli results in a pro-inflammatory profile characterized predominantly by M1 macrophages, in contrast to the M2 macrophages found in stable adipose tissue. A parallel is also observed between CD8 and CD4 T lymphocyte infiltration (Th1, Th17, and Th22), in which T regulatory lymphocytes are displaced. Consequently, there is a lack of tolerogenic tissue response and a high local inflammatory response. Moreover, neutrophil migration appears to be influenced by IL-17 production [129,130]. B cells’ role seems to depend upon the infiltration of cells and the presence of T lymphocytes. The polyclonal stimulation of B cells may generate the formation of autoantibodies in the tissues [107,108]. The inhibition or resolution of the inflammatory response passes by the inhibition of IL-1β signaling by the production of IL-1RA and the secretion of TGFβ [129,130]. The process can be facilitated by the production of IL-10 by the local immune cells. It is also possible that the secretion of lipids from activated adipocytes modulates the inflammatory response. The saturated lipids may enhance the production of lipid intermediates, facilitating the inflammatory process [131]. On the contrary, the presence of ω3 state fatty acid resolves inflammation [132,133].

It is important to note that the inflammatory response in adipose tissue is not uniform across different types [10]. Specifically, the increase in visceral adipose tissue correlates more strongly with insulin resistance than the increase in subcutaneous adipose tissue [45]. This suggests that subcutaneous fat may be less stable and more inflammation-resistant than visceral fat. Additionally, alterations in energy demands or surgical interventions may affect the dynamics of visceral adipose tissue, potentially enhancing the local immune cell response by stabilizing the inflammatory environment [45,46].

Cellular senescence is characterized by an irreversible arrest of the cell cycle, typically initiated by various forms of cellular stress [134,135,136]. Cells that undergo senescence exhibit a senescence-associated secretory phenotype (SASP), which includes the secretion of pro-inflammatory cytokines, chemokines, growth factors, and proteases [136,137,138,139]. Immunosenescence represents a complex process associated with aging, involving significant changes in the architecture and functionality of immune organs, ultimately leading to compromised innate and adaptive immune responses [136,137,138,139]. Although the precise molecular and cellular mechanisms are not fully elucidated, several prominent features of immunosenescence have been identified [136,137,138,139,140,141]. These include thymic involution, dysfunction of hematopoietic stem cells, disruption of T and B lymphocyte homeostasis, chronic low-grade inflammation (often referred to as inflammaging), accumulation of senescent cells, impaired antigen response, mitochondrial dysfunction, genomic instability, and enhanced stress responses [136,137,138,139,140,141].

Obesity contributes to the accelerated aging of adipose tissue, promoting the premature senescence of adipocytes [136,137,138,139,140,141]. Senescent adipocytes release increased quantities of free fatty acids (FFAs) and adipokines, including leptin, TNF-α, and IL-6 [136,137,138,139,140,141]. The SASP phenomenon can potentially induce senescence in adjacent tissues, particularly within the immune system [136,137,138,139,140,141]. Furthermore, adipose tissue in individuals with obesity is markedly infiltrated by B cells [139]. These adipose tissue-resident B cells are either recruited or activated by the byproducts of altered lipolysis, and the adipokines are secreted by expanding adipose tissue as they express the corresponding receptors [139]. Interestingly, Valentino [142] and coworkers analyzed the role of autoantibody formation, cell senescence, and aging, providing a fascinating insight into the process and suggesting possible therapeutic targets. The roles of B1 and B2 in adipose tissue, normal immune response, and autoimmunity are still under research.

### Adipocyte-Derived Extracellular Vesicles

Circulating extracellular vesicles (EVs) are recognized as significant mediators of cell-to-cell communication and the exchange of biological messages [143,144]. These lipid bilayer nanoparticles range in size from 50 to 1000 nanometres and can be released by nearly all cell types [143]. They are present in various body fluids, including blood, saliva, urine, breast milk, and amniotic fluid. Notably, adipose tissue serves as a crucial source of circulating EVs. The research indicates that individuals with obesity generally exhibit elevated levels of EVs in their serum compared to those without [144,145]. The underlying cause of this increase remains unclear; however, it has been suggested that fatty tissue in the context of obesity may produce EVs at a higher rate or exhibit a reduced capacity for EV elimination by the liver. Importantly, interventions, such as bariatric surgery or caloric restriction, have been shown to decrease the number of circulating EVs, implying that a reduction in adipose tissue mass correlates with diminished EV secretion [145]. Recently, EVs have been acknowledged as effective messengers for intercellular communication. Emerging evidence highlights that adipose-derived EVs play a vital role in the interactions among macrophages, adipocytes, and adipose tissue-derived stem cells, significantly influencing immunometabolism in healthy and obese states [142,143,144,145].

The significance of microRNAs (miRNAs) in the context of adipose tissue and inflammation is noteworthy. EVs derived from adipocytes and immune cells are instrumental in differentiating various cell types within the tissue. Rakib and colleagues [146] reviewed a potential mechanism involving miRNA-34a, which is secreted by activated adipose tissue and functions to inhibit the transcription factor KLF4, thereby obstructing the transformation of M2 macrophages [146]. Conversely, miRNA-326 secreted by M1 macrophages enhances the expression of RORC2, resulting in the upregulation of Th17 cells, which facilitates the inflammatory response [146].

Additionally, the miRNA-34 family is implicated in cellular senescence. In conjunction with miRNA-155, miRNA-34 contributes to telomere shortening [146]. Furthermore, miRNA-146 and miRNA-181 promote cellular senescence, while miRNA-335 is involved in “inflammaging” [146]. These molecular mechanisms lead to an exacerbated local inflammatory response due to increased cell senescence and mortality, which can, in turn, promote peripheral inflammation. In conclusion, EVs derived from adipose tissue may be pivotal in mediating tissue and multi-organ senescence and contributing to peripheral inflammatory responses.

Current investigations involving seemingly healthy, obese individuals indicate notable variations in the size, quantity, and composition of EVs. Compared to non-obese individuals, these variations appear to correlate with specific metabolic parameters, such as glucose levels, insulin sensitivity, and serum lipid profiles. Considering these observations, it is hypothesized that EVs play a significant role in progressing metabolic and cardiovascular complications associated with obesity [147]. However, the precise mechanisms underlying this process remain to be elucidated [148].

## 4. Obesity and Infectious Diseases

The research regarding the interaction between obesity and various infectious agents remains contentious and presents a highly intricate scenario [148,149]. The increased susceptibility to numerous types of infections among individuals with obesity is not yet fully understood. Obese individuals can have micronutrient deficiency [150], which may affect their response to viral infections, as observed in SARS-CoV-2 [151,152]. For example, the Edmonton obesity staging system reported that the impairment of vitamin D nutritional status and metabolic profile was associated with worsened obesity [153,154]. Thus, vitamin D deficiency may be linked to the impaired immune response observed in SARS-CoV-2 infection in obese individuals, and EVs may play an essential role in the severity of the disease.

Additional cofactors frequently linked to obesity may indirectly contribute to the development or exacerbation of infectious diseases, even in the absence of a clear causal relationship [148,149]. These cofactors encompass modifications in respiratory physiology, skin, and soft tissue integrity changes; co-morbidities, such as type 2 diabetes mellitus and cardiovascular disease; pharmacological interventions; and inadequate antimicrobial treatment [148,149].

The outcomes of infections in obese individuals and animal models appear to vary depending on the extent of the infection, likely due to differential impacts on the metabolic pathways of immune cells [10]. Obesity is a significant disruptor of bodily homeostasis, leading to alterations in immune metabolic pathways, often resulting in a diminished protective immune response to infections. The specific modifications in immune response due to obesity are still being fully elucidated. As documented in Table 2, the decreased production of IFN type I and the high secretion of IL-1RA may be related to a reduced effective response in obese individuals [31,36]. Pugliese et al. [149] analyzed the most relevant infection sites for obese patients. Upper respiratory tract infections are most commonly associated with pharyngitis, sinusitis, laryngotracheitis, lower respiratory infections, bronchitis, bronchiolitis, and pneumonia. Since sleep apnea in obese individuals increases, the risk of respiratory infections increases [155]. Hypoxia may jeopardize the response to treatment. Then, urinary tract infections (cystitis, urethritis, and pyelonephritis), skin infections (high incidence of cellulitis, candida, erysipelas, and onychomycosis), and surgical-site infections.

A growing body of research demonstrates that women living with HIV experience a significantly elevated risk of developing metabolic disorders in comparison to their male counterparts [156]. These metabolic disorders encompass weight gain and obesity, type 2 diabetes mellitus, dyslipidemia, bone loss, and sarcopenia. Conversely, men diagnosed with HIV exhibit a greater susceptibility to conditions such as hepatic steatosis and fibrosis [156].

Table 5 summarizes the relationship between viral infections, adipose tissue, obesity, and interferon response in different reports [157,158,159,160,161,162,163,164,165,166,167,168,169,170,171]. The table aims to provide the reader with insights concerning the pathogens, the possible role of adipose tissue to be altered upon the inflammatory response generated by the infection, and the effect of the virus on the IFN response. The decreased antiviral response and chronicity can be potentiated by the intermediates generated by adipose tissue as part of the response to the infection; however, the role of adipose tissue as a reservoir of virus or the role of adipose tissue on viral escape cannot be overlooked. More research is required in this area.

Hornung et al. [172] revised different in vivo and in vitro models to study the role of adipose tissue in bacterial and viral infections; however, most of the effort involved murine models, which are informative but do not necessarily follow the same response as humans. Hales and coworkers [173] explored the role of leptin in *Streptococcus pneumonia* infections and the difference between the results in humans and mice, showing the importance of the hormone in immune cell activation and response.

However, critical elements have received insufficient attention in the existing literature. The first pertains to the increase in cell death among underweight individuals with sepsis in the intensive care unit and the differences in gender responses observed in overweight and obese populations [174]. These observations prompt new inquiries regarding the incidence of infections and the immune response in obese individuals and probably better therapeutic strategies to protect individuals from severe infections.

## 5. Impact of Obesity on Vaccination Response

While substantial progress has been made through vaccination in protecting against infectious diseases, specific populations seem to exhibit suboptimal responses to these interventions, increasing the vulnerability of these groups to vaccine-preventable illnesses. Obesity may significantly influence vaccine immunogenicity and efficacy, potentially exacerbating the likelihood of an inadequate immune response [4,5,6]. The negative impact of obesity on immune system functionality raises concerns regarding the effectiveness of the vaccine within this demographic. The initial studies indicating a potential correlation between obesity and compromised immune response to vaccinations were published in 1985, focusing on a cohort of obese hospital employees who demonstrated a poor response to the hepatitis B vaccine [175]. Therefore, it is imperative to explore strategies to enhance the protection of this at-risk population.

### 5.1. Inactivated or Subunit Vaccines

Inactivated vaccines consist of whole-cell formulations containing a version of the entire viral or bacterial pathogen that has been rendered inactive. Conversely, subunit vaccines comprise only specific components of the virus or bacteria that contain the necessary antigens to elicit an immune response while excluding all other molecular elements present in the pathogen [176]. Inactivated or subunit vaccines do not resemble the live pathogen, and therefore, the immune response is usually defined by antibody production and neutralizing antibodies the critical endpoint to protect against the infection [176].

Obesity may impede an individual’s capacity to generate an effective immune response to vaccination or infection, a phenomenon attributable to increased body fat and elevated leptin levels. Callahan et al. [177] undertook a comprehensive analysis of pooled data from three independently conducted, NIH-supported phase 2 clinical trials assessing monovalent, unadjuvanted, split-virus pandemic H1N1 vaccines administered at eight Vaccine and Treatment Evaluation Units (VTEUs) between August 2009 and March 2010. One trial was conducted with children and adolescents (6 months to 17 years old), utilizing the Sanofi Pasteur vaccine. The other two trials, designed with identical methodologies, recruited non-pregnant adults (age ≥ 18 years) and used vaccines manufactured by Sanofi Pasteur or CSL Biotherapies. Participants were randomly assigned to receive two intramuscular injections, which contained either 15 or 30 International Units (IUs) of hemagglutinin (HA), measured by high-performance liquid chromatography, administered 21 days apart. The final potency evaluation of the Sanofi Pasteur vaccine, conducted using the single radial immunodiffusion (SRID) assay, indicated an HA content of 22–25 IU for the 15 IU dose (analyzed across two different batches) and 47 IU for the 30 IU dose. Among adult subjects, nearly 30% were classified as obese or morbidly obese, 37% as overweight, and only 1% as underweight. The findings concluded that a single dose of the vaccine prompted higher hemagglutination inhibition geometric mean titers (GMTs) on day 21 in obese adults compared to individuals in other BMI categories [175].

Clarke et al. [178] examined the effects of obesity on responses to the quadrivalent influenza vaccine in children. This study enrolled children classified as having obesity (BMI) ≥ 95th percentile for age and gender) and those without obesity (BMI < 95th percentile). Blood samples were collected before vaccination and at one and six months post-vaccination to evaluate antibody responses utilizing the hemagglutination inhibition assay. The immunogenicity of the vaccine was compared across the two groups of children. Both groups, those with and without obesity, demonstrated robust and sustained antibody responses to the tetravalent influenza vaccine six months post-vaccination. Sheridan et al. [179] reported that a higher BMI initially correlated with an enhanced antibody response following vaccination with the inactivated trivalent influenza vaccine. Nonetheless, twelve months after vaccination, a higher BMI was associated with a more significant decline in antibody levels and a decreased presence of specific CD8+ T lymphocytes and IFNγ production in obese individuals [179].

Huang et al. [180,181] conducted a study involving children aged 8 to 18 years who had completed their routine childhood immunizations. Serum samples were analyzed using ELISA to assess antibody levels against diphtheria, tetanus, *Haemophilus influenzae* type B, and *Streptococcus pneumoniae*, in addition to measuring serum HbA1c levels. BMI percentiles and HbA1c levels were utilized as continuous variables about antibody titer levels. The study revealed that 43% of the children had a BMI at or above the 95th percentile (n = 69). A notable negative correlation was observed between BMI and the antibody titers for pneumococcal, diphtheria, and tetanus vaccines, with a significant correlation identified explicitly for the *S. pneumoniae* serotype P3 titer (*p* = 0.037). The findings indicate increased BMI and HbA1c levels are associated with lower overall vaccine titers. Additionally, the study highlighted that obese children (BMI ≥ 95%) exhibited a higher likelihood of having impaired pneumococcal titers compared to their non-obese counterparts (BMI 25–94%) in a prospective, population-based cohort study [180,181].

Zimmerman and Curtis [182] analyzed critical elements that affect vaccine response. Several aspects, such as environmental factors, infection, viral, bacterial, and parasitic, negatively impact the immune response against inactivated and subunit vaccines. Consequently, the low titer of antibodies and the low memory response may require revaccination or the design of new vaccines with a higher immune response activation.

### 5.2. Live-Attenuated Vaccines

Vashishtha and Kumar [183] reviewed the efficacy of various vaccines regarding their ability to prevent infection and disease and their longevity of protection. Live-attenuated vaccines, such as those for measles, rubella, and yellow fever, provide long-lasting immunity. In contrast, vaccines for hepatitis A, BCG, varicella zoster, and mumps offer moderate protection, while vaccines for dengue, herpes, and rotavirus yield only short-term immunity [183]. The variability in the protective efficacy of vaccines is linked to the immune system’s ability to recognize antigens, which various conditions may influence, not only obesity, as elucidated by Zimmerman and Curtis. [182]. Obesity is not a critical factor in live-attenuated vaccine efficiency [182,183].

Dumrisilp and colleagues [184] conducted a prospective study with children aged between seven and twenty-five years from Bangkok and the obesity outpatient center at King Chulalongkorn Memorial Hospital. The enrolled individuals, 212, were vaccinated with MEVAC™-A (hepatitis A live-attenuated virus). Blood samples were collected to assess the levels of anti-HAV antibodies one day before vaccination and 8–9 weeks post-vaccination. According to prior studies, an anti-HAV IgG titer of 20 mIU/mL is deemed seroprotective. Statistical analysis revealed that a single administration of the live-attenuated hepatitis A vaccine is both safe and highly immunogenic in subjects classified as either underweight/normal weight or overweight/obese during the brief follow-up period. Truncal obesity and female gender were identified as factors associated with an enhanced immune response; however, no significant differences in anti-HAV titers were observed between the non-obese and obese groups, nor between the child and young adult cohorts. It is important to note that the duration of follow up for evaluating safety and immunogenicity was relatively brief, limited to only nine weeks [184]. Thereafter, Soponkanabhorn et al. [185] conducted a retrospective study utilizing blood samples from Dumrisilp et al. [184]. The results of this study suggest that obesity does not affect the short-term cellular immune response to HAV live-attenuated virus vaccination. However, this clinical trial had several limitations, one of which was the absence of data regarding cardiometabolic risk factors, specifically in obese participants, and the other limitation of the clinical trial is the relatively long interval between vaccination and subsequent immunogenicity testing. The long period between vaccination and evaluation may explain the absence of substantial improvement in vaccine-induced cell-mediated immunity in most participants [184,185].

Fonzo et al. [186] conducted a clinical trial involving 2185 students at the School of Medicine, University of Padua (815 males and 1370 females). The objective of this study was to examine the relationship between BMI and current antibody levels following vaccinations for measles, mumps, and rubella (MMR) and a recombinant hepatitis B virus (HBV), which were administered during childhood. The BMI was classified based on the World Health Organization criteria. There is no significant association between BMI and the persistence of immune response after HBV and MMR vaccinations. Furthermore, no noteworthy sex-related differences were observed in the results [186].

SARS-CoV-2 inactivated vaccines decreased antibody titer production in individuals with severe obesity and BMI ≥ 40 [187]. A study using recombinant SARS-CoV-2 vaccine and inactivated influenza virus generated similar results; a low reaction was observed in obese individuals [188]. Furthermore, in a small observational study by Frasca and coworkers [189], the authors showed differences in B defects in obesity and an improvement when individuals successfully lost weight. These results suggest that weight reduction may decrease B lymphocyte impairment. However, these results should be analyzed with care since vaccination routes, schemes, and doses were probably inappropriate for the obese population, especially morbid obesity. Well-defined critical trials should investigate the impact of overweight and obesity, considering the endocrinological response, gender, and age.

### 5.3. RNA and Recombinant Vaccines

Messenger RNA (mRNA) vaccines prompt the body’s cells to make a specific protein fragment to serve as an antigen to generate an antibody response [190]. On the contrary, recombinant protein vaccines are created through conventional genetic engineering based on targeted pathogens’ proteins that can stimulate the immune system [191]. Unlike other vaccine types that utilize viral genetic material or vectors, recombinant protein vaccines use only antigenic proteins [191]. In both cases, generating specific and neutralizing antibodies is the primary goal.

Clinical trials examining BMI and central obesity have indicated that individuals with obesity exhibited lower antibody titers in response to vaccination compared to those of a healthy weight [4,5,6,192]. This observation highlights a potential early decline in vaccine-induced antibody levels correlated with higher obesity rates. Consequently, the anticipated protective effects of SARS-CoV-2 vaccination may be diminished in individuals with obesity relative to their healthy-weight counterparts [4]. The results were challenged by other researchers [7]. The discrepancies are due to the cohort analysis and the possible involvement of factors besides obesity, as pointed out by Zimmerman and Curtis [182]. No specific data of the cohorts were likely obtained from the reports that impaired endocrinological responses are responsible for the decreased responses reported in individuals with BMI.

Ou et al. [193] performed a meta-analysis of the literature, examining antibody responses to COVID-19 vaccinations among individuals with and without obesity. This meta-analysis incorporated the findings from eleven studies, five of which provided absolute values of antibody titers for both the obese and non-obese groups. The results indicated that the obese population exhibits a statistically significant association with lower antibody titers following COVID-19 vaccination [193]. Similarly, Faizo et al. [194] reported comparable findings. Their study analyzed sera from a vaccinated low number of obese individuals (n = 73) alongside controls with a normal BMI (n = 46). The samples were analyzed for total anti-S protein and neutralizing antibodies. Additionally, a nucleocapsid ELISA was employed to differentiate between immunity obtained solely through vaccination and acquired through a combination of vaccination and recovery from infection. This study also revealed a decrease in vaccine-induced neutralizing humoral immunity among obese participants, a phenomenon observed regardless of gender, previous infection recovery, and the time elapsed since the last vaccination [194]. Even though the reports suggest a decrease in antibody response in obesity, well-controlled assays are essential. Several factors, such as vitamin D deficiency and gender, endocrine function, environmental factors, vaccine dose, adjuvants, route of administration, and malnutrition, should be analyzed carefully.

### 5.4. Heterleogous Vaccination: COVID-19 Vaccines

During the COVID-19 pandemic, several countries considered using a boosting vaccine that was different from the first vaccine treatment. Shaw and coworkers [195] conducted a study investigating the durability of immune response to viral vector, mRNA, and protein-based COVID-19 vaccine platforms used in homologous and heterologous priming combinations, which will guide future vaccine platform selection. The study was a single-blinded trial in which adults ≥ 50 years old, previously immunized with single-dose “ChAd” (ChAdOx1 nCoV-19, AZD1222, Vaxzevria, and Astrazeneca) or “BNT” (BNT162b2, tozinameran, Comirnaty, and Pfizer/BioNTech), were randomized 1:1:1 to receive a second dose 8–12 weeks later with either the homologous vaccine or “Mod” (mRNA-1273, Spikevax, or Moderna) or “NVX” (NVX-CoV2373, Nuvaxovid, or Novavax). Immunological follow up and safety monitoring took place over nine months. Antibody and cellular assays were analyzed for individuals without evidence of COVID-19 infection.

The results show that heterologous priming schedules utilizing ChAd vaccines demonstrate a more significant immunogenic response over time than ChAd/ChAd regimens. Similarly, treatment schedules initiated with BNT vaccines followed by a second dose of either mRNA vaccine exhibit superior long-term immunogenicity relative to BNT/NVX combinations [195]. The amount of neutralizing antibody was higher with the BNT/Mod combination. The authors concluded that mixed vaccination schedules incorporating novel vaccine platforms, particularly those deployed during the COVID-19 pandemic, indicate that heterologous priming may be a viable option to consider earlier in future pandemic responses [195].

Interestingly, the authors [195] reported that the immunogenic response to NVX was significantly decreased in obese individuals, and T-cell responses in BNT/BTN negatively correlated with BMI. At the same time, the BNT/Mod showed the reverse trend. Even though no apparent statistical differences were recorded in gender, female participants responded more than males in all scheduled vaccinations except BNT/NVX, and the decay of immune response was less in females with the ChAd/NVX scheme than in males. Sheehan et al. [196] showed a decrease in neutralizing antibodies following multiple doses of BNT162b2, which partially supported the results of Shaw et al. [195]. These results suggest that the vaccination schemes are critical, and that heterologous immunization may decrease the factors affecting vaccine response.

### 5.5. Gender, Thyroid Function, and Vaccine Response

Sex-related differences in immune cell function are linked to the expression of genes located on the X or Y sex chromosomes and variations in autosomal gene expression within immune cells that arise from several hormone receptor signaling pathways and epigenetic modifications. These differences in immune function are dynamic, evolving throughout the lifespan and during various reproductive stages [56,63,197]. Gender-related variations represent a significant potential source of variability that influences immune response to vaccination (analyzed in Table 3). Females and males exhibit distinct differences in their innate, humoral, and cell-mediated immune responses to vaccines. The female gender has been reported to have a higher incidence and severity of adverse effects following vaccination, including symptoms such as fever, pain, and inflammation [198].

Pregnancy can further modulate immune responses to vaccines. Following immunization against pathogens, such as influenza, yellow fever, rubella, measles, mumps, hepatitis A and B, herpes simplex virus type 2, rabies, smallpox, dengue virus, and SARS-CoV-2, neutralizing antibody responses in adult women may be up to twice as high as those observed in men [199]. Women are also more likely to experience severe adverse reactions, such as localized and systemic pain, inflammation, fever, and hypersensitivity reactions. It has often been suggested that the greater incidence of adverse events among women is attributable to sociocultural behaviors and habits rather than to biological differences, mainly since the data regarding adverse events are typically collected through passive reporting mechanisms [200,201].

Dissection of the human immune response to the gold standard vaccine (yellow fever virus 17D), composed of live-attenuated viruses, has been based on several systems biology studies to demonstrate that the transcriptional profiles of innate immunity genes, including TLR and interferon gene expression, immediately after vaccination with yellow fever virus 17D are predictive of subsequent immune responses [202]. Most adverse events following yellow fever virus vaccination reported to the Vaccine Adverse Event Reporting System (VAERS) between 2000 and 2006 were mild and self-limiting. As VAERS is a passive reporting system, it should also be noted that women are more likely to report adverse events than men [202]. In addition, the immune response to the yellow fever virus 17D vaccine was higher in females than in males, as has been described in several research studies for most vaccines, with some exceptions [203,204].

Pertussis infection is more common in women than in men. However, the variability in the sources of these observations makes it difficult to estimate the magnitude and consistency of sex differences by age. To assess this issue, Peer et al. [205] used meta-analytic methods to evaluate national pertussis incidence rates by sex and age group in nine countries between 1990 and 2017. The authors concluded that the excess incidence of pertussis among women, especially in infants and very young children, is unlikely to be due to differences in exposure [205]. Future studies should take sex into account to better understand the mechanisms affecting disease incidence, with possible implications for disease control.

Boef et al. [206] systematically re-analyzed childhood vaccination studies conducted in the Netherlands for sex differences in IgG responses. Six studies with IgG determinations in 1577 children after childhood pneumococcal vaccination (PCV7/PCV10/PCV13) and/or DTaP-IPV-Hib(-HepB) or DTaP-IPV preschool booster were included. For most vaccine antigens investigated, there were no consistent differences in vaccine-induced IgG levels and gender. Vaccine-induced pneumococcal IgG levels were slightly higher in girls, but only between the primary series and the 11-month booster. These results and similar reactogenicity and vaccine failure/efficacy support the consistent childhood vaccination schedule in the Dutch national immunization program [206].

The role of thyroid hormones in immune and vaccine response is not fully understood [207]. Recent studies using mRNA vaccine have shown that the response of patients with autoimmune thyroiditis is similar to that of controls; however, the vaccine may affect thyroid function [208]. On the other hand, the response to the same vaccine in patients with Hashimoto thyroiditis is higher than that of controls [209]. There are still many unanswered questions in this field related to sex and obesity, and it requires more research.

## 6. Microbiota

In recent years, several authors have postulated the possible role of microbiota in vaccine response [210,211]. Some have also added that sex hormones will modulate gut microbiota, so the vaccine’s efficiency may be related to this event [211]. Even though the conclusions of those reports were based on analyzing different cohorts from different areas of the world, most of the trials were performed with a limited number of individuals and with little information about the volunteers [208]. As pointed out by Syromyatnikov and coworkers [212], populations are heterogeneous, and the relationship of gut microbiota with a disease may depend on several factors: diet, may be affected by religion, environment, genetic background, and others. In the specific case of obesity, several bacterial families have been linked, depending on the country. In the USA, *Rummicocaceae* and *Oxolobacter* families have been associated with obesity; however, in other countries, *Firmicutes*, *Bacteroidota*, and *Clostridiales*, among others, have been considered relevant [212].

An analysis of the heterogeneous population in the United States reveals a high incidence of overweight and obesity, as indicated by the World Health Organization’s guidelines [213]. When further examined by race, it is evident that African Americans and Hispanic Latinos exhibit a higher prevalence of overweight and obesity in comparison to White and Asian Americans, the latter group reporting the lowest incidence rates [214]. Gender-specific data indicate that approximately 80% of African American women are classified as overweight or obese, with these individuals exhibiting a greater likelihood of obesity than their non-Hispanic White and Asian American counterparts, with respective rates of 50% and 90% [215,216]. Stanislawski et al. [215] report suggests that the correlation between lower alpha gut microbiota diversity and elevated BMI may be particularly pronounced among non-Hispanic White populations and individuals of higher socioeconomic status. In contrast, the relationship between increased relative abundance of *Prevotella*, low beta diversity in gut microbiota, and BMI appears more significant in Black and Hispanic populations [217].

Norton and coworkers [218] used the BNT162b2 COVID-19 mRNA vaccine to show similar immune responses to normal mice in a mouse model using antibiotic-depleted germ-free C57BL/6J mice. Moreover, the authors performed fecal transplants on the germ-free mice, showing a decreased response compared to the not-transplanted mice [218]. Even though this report is on a mouse model, it raises an interesting question regarding the connection between microbiota and immune response to vaccines.

Inulin treatment in kidney transplant patients with dysbiosis increased the amount of *Bifidobacterium*, which is associated with enhanced vaccine responses [219]. Despite this increase in *Bifidobacterium*, no differences were encountered in the in vitro neutralization of live SARS-CoV-2 virus at 4 weeks following a third vaccination [219]. It can be concluded that some of the previous assumptions have to be re-analyzed in well-controlled clinical trials.

The gut microbiota phenotypes associated with obesity may differ based on gender, race, ethnicity, and related factors such as dietary habits and socioeconomic status. Furthermore, microbiome studies often face limitations due to small sample sizes, which complicate exploring intricate interaction effects. Modifications in microbiota do not necessarily result in a more efficient immune response [220]. Significantly, non-White populations are greatly underrepresented in cohorts, which presents substantial barriers to fully understanding population-level patterns in the microbiome, obesity, gender, and vaccine relationships.

Recent studies have facilitated a preliminary understanding of the thyroid–gut axis, suggesting that intestinal microbiota and their metabolites may influence the thyroid gland directly or indirectly [221,222,223]. This influence may occur through mechanisms such as the uptake of intestinal microelements, the conversion and storage of iodothyronines, and the regulation of immune responses [221,222,223]. These findings provide valuable insights into the pathogenesis of thyroid disorders and potential clinical management strategies [221,222,223]. However, the existing research on the relationship between gut microbiota and thyroid function has only scratched the surface of this complex interaction. There remains a pressing need for more comprehensive clinical data and foundational experiments to clarify the specific relationships and mechanisms at play.

## 7. Limitations of the Studies Involving Overweight and Obesity

Overweight and obesity represent significant challenges in human healthcare [213]. Nonetheless, advancements in the scientific research and therapeutic interventions have been comparatively constrained. The insufficient correlation between animal models and human subjects is a primary obstacle. While murine models benefit from defined genetic backgrounds, established physiological responses, and simplified intervention and sample analysis methods, human participants exhibit high heterogeneity. This variability complicates sample collection and analysis, rendering interpretation of the data often challenging. Moreover, the conclusions drawn from clinical trials and meta-analyses are limited, given the many parameters that must be considered and usually are not reported in published articles.

Other limitations in vaccine studies include race, socioeconomic status, malnutrition, environmental conditions, and others, as described by Zimmerman and Curtis [182]. General conclusions based on partial information can lead to critical bias in the analysis of the results and, consequently, in the design of new vaccines or strategies to enhance vaccine efficiency.

## 8. Future Perspectives

Chronic unresolved systemic and adipose tissue inflammation significantly contributes to the onset of obesity-related cardiometabolic diseases. While pharmaceuticals targeting pro-inflammatory cytokines or inflammasome activation have received clinical approval, their widespread application is often limited by severe adverse effects, including weight gain and increased susceptibility to infections. These factors hinder their broader clinical implementation. There remains a pronounced gap in the availability of biomarkers that can effectively differentiate between acute and chronic inflammation and assess the functionality of distinct leukocyte populations. Developing such biomarkers would enhance personalized treatment approaches and facilitate the monitoring of therapeutic interventions. The resolution phase of inflammation is an active and regulated process governed by specialized pro-resolving mediators, which have demonstrated efficacy in alleviating obesity-related inflammation and systemic disease in experimental models. This area represents a significant opportunity for therapeutic advancement.

Recent technological advancements are facilitating the development of more effective and innovative vaccines and adjuvants. The primary objective of these efforts is to restore inflammatory and immune homeostasis while maintaining other essential physiological processes. This aim may be accomplished by enhancing leptin sensitivity through leptin-based therapeutic strategies, encompassing synthetic or modified leptin and pharmaceuticals that selectively target leptin-induced pathways [224,225].

The use of GLP-1 therapies for obesity has generated a new area of research. The GLP-1 receptor is a negative costimulatory receptor [226], which could lead to possible use in cancer and other diseases. Van Niekerk and coworkers [227] postulated in a recent review that the response to vaccines may be lowered due to the effect of the drug on immune cells; however, a system of delivery or modification of the pharmacological strategies may facilitate the therapeutic effect of the drug without affecting the immune response. There is still room for improvement in this area.

It is also critical to analyze the possibility of autoimmune disease in the process of adipose tissue dysregulation that could lead to a less effective response [142]. Prolonged SARS-CoV-2 infection and other chronic viral diseases [107,188,228,229] have been related to an increased incidence of autoimmunity. Conversely, the mechanism by which obesity is linked to the generation of autoimmune disease and circulating autoantibodies is still a matter of research.

Emerging technologies aimed at enhancing immune responses, including innovative adjuvants, advanced delivery systems, and alternative vaccine administration routes, have the potential to improve vaccine efficacy significantly [230]. For instance, intranasal or oral vaccines present a viable solution to the challenges posed by issues related to overweight and obesity. Another solution is nanotechnology to enhance antigen absorbance and distribution in the microenvironment to induce a better response [231]. In summary, a future solution in this complex field is highly possible. However, as shown by Shawn and coworkers [195], simple solutions, such as heterologous vaccinations, may solve critical issues, especially in underdeveloped countries where vaccination with new technologies may not be affordable.

## 9. Conclusions

**Figure 3:**
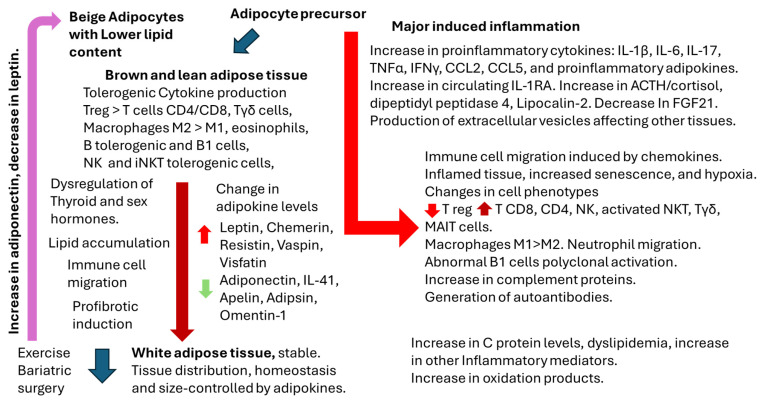
A summary of the points analyzed in this review. The description of immune cells involved in brown and lean adipose tissue, the process that induces the formation of white adipose tissue, and the effect of exercise and bariatric surgery. On the right is the process related to inflammation, which involves significant metabolic and immune response-related changes, including autoimmunity. Subclinical inflammation has been described in overweight and obesity (metabolic syndrome) and has been involved in several diseases.

In the current review, summarized in Figure 3, we have outlined the most significant aspects of adipose tissue physiology, the influence of sex hormones, adipokines, associated cytokines, and the presence of local immune cells under normal conditions and during inflammatory responses. Additionally, we have examined the role of sex hormones in these processes. Despite our thorough investigation, we have only partially addressed the critical components necessary for understanding the reasons behind the diminished vaccine responses observed in specific obese individuals. Further research is essential in this domain, particularly considering the growing prevalence of overweight and obesity globally.

From our perspective, resting adipose tissue is characterized by a tolerogenic environment, which does not influence insulin resistance or lipid metabolism and, consequently, does not impact the immune response to vaccinations. In contrast, subclinical inflamed adipose tissue, marked by immune cell migration and the production of local and peripheral pro-inflammatory cytokines and adipokines, does influence responses to infections and vaccines. To better delineate this difference, it is essential to identify specific biomarkers that account for variables such as gender, sex hormones, the hypothalamic–pituitary–adrenal axis, thyroid hormones, nutritional factors, and both genetic and epigenetic modifications.

The vaccines developed to combat the SARS-CoV-2 virus have introduced a novel framework for analyzing the vaccination process. The heterologous vaccination results have provided an intriguing possibility of enhancing immune response and probably decreasing unwanted side effects. Nevertheless, further research is essential to understand the limitations of these vaccines, particularly regarding their safety, efficacy, and overall effectiveness within the population.

## Figures and Tables

**Table 1:** Adipokines and their role in inflammatory response.

Adipokine	Pro-Inflammatory	Anti-Inflammatory	Reference
Adiponectin	No	Yes	[12,13]
Adipsin (complement factor-D)	No	Yes	[14,15]
Apelin	No	Yes	[16]
Chemerin	Yes	No	[17]
Leptin	Yes	Yes	[18,19]
Meteorin like (IL41)	No	Yes	[20,21]
Omentin-1	No	Yes	[22]
Resistin	Yes	No	[23]
Vaspin	Yes	Yes	[24,25]
Visfatin	Yes	No	[26]

**Table 2:** Other cytokines and factors involved in adipose tissue responses.

	Effect	Reference
CCL2 (MCP-1)	Monocyte migration to adipose tissue.	[27]
CCL5	Monocyte migration to adipose tissue.	[28]
CCL22	Thermogenesis induction.	[29]
IL-6	Local activation of immune cells. Metabolic dysregulation.	[30]
IFN	IFNα induces apoptosis in adipocytes. IFNβ regulates metabolism. IFNγ pro-inflammatory response; reduction in adipose tissue. IFNλ1 enhances inflammatory response. IFNτ reduces inflammatory response.	[31]
TNFα	Activation of tissue immune cells. Metabolic dysregulation.	[32,33]
IL-1 and IL-RA	IL-1 α hypertrophy of white adipose tissue. IL-1 β promotes adipogenesis in murine and human adipose-derived stem cells. IL-RA is upregulated in white adipose tissue, and high circulating levels in obesity.	[34,35,36,37]
Dipeptidyl peptidase 4	Plays a role in metabolic homeostasis and inflammatory response. Inhibition of the enzyme, combined with metformin, induces a significant decrease in visceral adipose tissue.	[38]
Fibroblast growth factor 21	Anti-inflammatory.	[39]
Retinol binding protein 4	Induction of inflammatory response. Inhibition of insulin signaling.	[40]
Lipocalin-2	Produced by white adipocytes. Increases adipose tissue. Involved in neutrophil chemoattraction.	[41,42]
TGFβ	Involved in tissue fibrosis and insulin resistance.	[43]

**Table 3:** Effect of estrogen, progesterone, androgens, and thyroid hormones on immune cells.

Immune Cells	Estrogens	Progestins	Androgens	Thyroid Hormones
Monocytes/macrophages	Inhibit pro-inflammatory cytokines. Increase phagocytosis	Inhibit inflammatory response and inhibit TLR4 and TLR9 activation	Enhance macrophage migration. Anti-inflammatory response	Increase phagocytosis (T3/T4). Increase M1 and decrease M2 differentiation (T3)
Dendritic cells	Promote cell differentiation. Promote pro-inflammatory cytokine production. Enhance T-cell activation	Decrease secretion of pro-inflammatory cytokines	Decrease pro-inflammatory cytokine production. Decrease T-cell stimulation	Promote maturation (T3/T4). Pro-inflammatory role (T3)
Neutrophils	Enhance cell activation and chemotaxis	Inhibition of neutrophil activation	Inhibition of neutrophil activation	Increase in oxidative burst and phagocytosis (T3/T4).
Mast cells	Increased inflammatory response	Decreased inflammatory response	Anti-inflammatory response	Mast cells store T3 and may impact thyroid function. T3 activates mast cells
Eosinophils	Enhanced cell activation	Decreased cell activation	No or low response	Not well defined. Activated cells affect the thyroid gland
NK cells	Activate NK cells	Modulate NK activity	No main effect on NK cells	Increased NK cytotoxic activity (T3/T4)
NKT cells	Decreased stimulation	Decreased stimulation	No response	No thyroid-stimulating hormone receptor is present
T γδ cells	Induce production of IL-17 and promote an increase in Th17	Tolerogenic responses	Induce cell activation	Not well defined. Activated cells may affect the thyroid gland
T cells	Increase in Th1 and Th17	Increase in Th2 and T reg cells	Decrease in Th17 cells	Increase in proliferative response and cytotoxicity
B cells	Increase the production of all types of antibodies, including IgE	Increase the production of IgG and IgA	Decrease in IgG secretion	Increase in proliferative and lymphopoiesis No defined role in antibody production

Table legend. The information presented is based on the references for sex hormones [56,57,58,61,62,63,71,72,73,74] and thyroid hormones [70,75,76,77,78,79,80,81].

**Table 4:** Summary of the effects of immune cells and mesenchymal stem cells on adipose tissue.

Cell Type	Effect	Reference
Neutrophils	Retain phagocytic activity, increase basal superoxide, and chemotaxis. Absolute neutrophil counts and neutrophil to lymphocyte ratio may indicate adipose tissue inflammation. Relationship of microbiota with neutrophil infiltration in adipose tissue.	[96,97,98]
Eosinophils	Protect adipose tissue from inflammation.	[99]
Mast cells	Mast cells are activated in human adipose tissue and localized preferentially in fibrosis depots.	[100]
Macrophages	M2 macrophages in lean tissue and M1 in inflammatory tissue.	[101]
iNKT cells	In lean adipose tissue, they can be activated by CD1 and can incorporate lipids, generating a local inflammatory response.	[91,103]
NK	Present in adipose tissue. Tolerogenic response in adipose tissue? Different responses depending on gender.	[104,105]
Tγδ	Inhibit inflammatory response.	[106]
B cells	Dysfunctional B cells in obese individuals. The lean adipose tissue contains B regulatory and B1 cells. B1 cells produce IgM antibodies for primary innate immunity. B2 cells usually generate protective antibodies in lymphoid organs. However, they participate in local inflammation and promote insulin resistance after migrating to white adipose tissue.	[107,108,109]
Th1 cells	Promote obesity-associated inflammation.	[108,111]
Th2	Stabilize adipose tissue and induce M2 polarization. A decrease in Th2 cells in the tissue is due to increased local IFNγ and inflammation.	[108,111]
Th17	Pro-inflammatory role. Related to IL-23 secretion in adipose tissue.	[112,113]
Th22	IL-22 is produced by innate lymphocyte cells upon tissue inflammation. It is related to insulin resistance.	[114]
CD8 cells	Cytotoxic response. Adipose tissue inflammation. Tissue remodeling.	[115,116]
Mucosal-associated invariant T (MAIT) cells	Secrete IL-17, inducing local tissue inflammation.	[117,118]
T follicular (TF) cells. TFh helper and TFreg regulatory cells	Modulate the response of B cells in adipose tissue. Impairment of TF regulatory cells is related to autoimmunity.	[119,120]
Follicular B cells	In adipose tissue, they induce inflammation depending on the cytokine milieu. Mesenchymal adipose stem cells induce the expansion of IL-10-producing B cells—possible role in autoimmunity.	[121]
Mesenchymal stem cells	Anti-inflammatory in the presence of Treg and Th2 milieu. Pro-inflammatory in the presence of inflammatory cytokines.	[122,123]

**Table 5:** Viral infection, adipose tissue involvement, and IFN responses.

Virus	Adipose Tissue Involvement	IFN Responses	Reference
Adenoviruses	Yes	Suppression. Chronic infection. Obesity-induced viral infection?	[156]
Arboviruses	Yes	Suppression. Chronic infection	[157]
Herpesviridae	Yes	HSV-1 suppression through miRNA CMV-multiple antagonistic mechanisms	[158]
Slow virus (Prion)	Yes	Inhibition of IFN signaling	[158]
Dengue	Yes	Inhibition of INF signaling	[159,160]
Papillomavirus	Yes	IFN signaling decreased	[161,162]
HCV	Yes	Antagonism of IFN signaling. Chronicity	[163]
HIV	Yes	Antagonism of IFN signaling. Chronicity	[164]
RSV	Yes	Inhibits IFN signaling	[165,166]
Coronavirus	Yes	IFN signaling is inhibited	[167,168]
Influenza	Yes	IFN signaling is inhibited	[169]
Hepatitis B virus	Yes	IFN response impaired	[170]
